# Extraction, separation and characterization of endotoxins in water samples using solid phase extraction and capillary electrophoresis-laser induced fluorescence

**DOI:** 10.1038/s41598-017-11232-x

**Published:** 2017-09-07

**Authors:** Fun Man Fung, Min Su, Hua-tao Feng, Sam Fong Yau Li

**Affiliations:** 1 Department of Chemistry, National University of Singapore, 3 Science Drive 3, Singapore, 117543 Singapore; 2 Institute for Application of Learning Science and Educational Technology (ALSET), University Hall, Lee Kong Chian Wing UHL #05–01D, 21 Lower Kent Ridge Road, Singapore, 119077 Singapore; 3 NUS Environmental Research Institute, National University of Singapore, T-Lab Building, 5A Engineering Drive 1, Singapore, 117411 Singapore

## Abstract

This study focuses on one of the key environmental threats, endotoxins, also known as lipopolysaccharides (LPS). A capillary electrophoresis method in combination with laser induced fluorescence (LIF) detection was developed for the analysis of endotoxins from 16 different bacterial strains. LPSs were derivatized with the amino-reactive fluorescent dye, fluorescein isothiocyanate (FITC), separated by capillary zone electrophoresis (CZE) under the optimized conditions with the use of 50 mM sodium tetraborate buffer (pH 9.30), and detected by LIF detector. To improve the sensitivity of CZE-LIF detection for the determination of trace amounts of endotoxins and to remove possible interference materials in environmental samples, a solid phase extraction (SPE) pre-concentration technique was applied successfully. The SPE targeted at polysaccharide moieties of LPSs and showed LPS enrichment effects too. CE migration time could also reveal the O-antigen chain lengths of LPSs. This CE method and SPE pretreatment showed linearity at 99.84%, and repeatabilities at 8.44% and 11.0% for endotoxins from *E. Coli* O55:B5 and *E. Coli* O26:B6. The limit of detection (LOD) could reach around 5 ng/mL at optimized condition. The method was applied successfully to the determination of LPS levels in tap water and wastewater, and demonstrated sensitive, reproducible and reliable results.

## Introduction

Endotoxins, also called Lipopolysaccharides (LPS), is the major constituent of the outer membrane of gram-negative bacteria^1^. The outer membrane serves not only as a barrier for environmental stresses and various toxic substances, such as antibiotics, but also as nutrient transporter^2^. The term LPS usually refers to the purified form; while endotoxin refers to the LPS attached on the bacterial membrane^3, 4^. LPS molecules which constitutes about 3.6% of a bacterial cell, occupy approximately three quarters of the surface area of the outer membrane of *Escherichia coli* ^4, 5^. LPSs are released from the bacterial surface during growth, cell division and lysis^1, 6^. As such, endotoxins are universal toxic inflammatory agents in the environment.

Endotoxins are toxic because they can influence many cellular and humoral mediated systems^7, 8^. LPS in the infected host’s blood stream induces high fever, leukocytosis, intravascular coagulation, multi organ failure, septic shock and even death^6, 9^. Even 1–10 ng (10–100 EU)/kg body weight (intravenously) of endotoxin can induce fever^10^. It is also found that endotoxins can augment the toxicity of some chemicals such as microcystins^11, 12^. Endotoxins have been considered as the main cause of pyrogenic reactions that might take place during the biotherapeutics administration^13^. In the Pharmacopoeia of the United States, China and the United Kingdom, a limit of 0.25 EU/mL has been established for injection water^10^. Four separate studies had indicated that endotoxin levels in surface water range from <1 to 1049 ng /mL^10^. So far, the risks associated with endotoxins in drinking water are not well-quantified. To date, there have been very few publications about the endotoxin levels in drinking water supplies, and no clear guideline values for endotoxin concentration have been established yet.

Used water treatment and reuse are extremely important in the sustainable development of water resources^14, 15^. However, large numbers of microorganisms are likely to be present in used water and secondary effluents. Therefore, removing these substances in reclaimed or reused water is extremely important^16^. During water treatment, the monitoring of endotoxin concentrations can promote the safety level. Endotoxin as typical toxic chemicals generated by microorganisms is one of the emerging contaminants. It has reportedly shown high activity in water treatment plant effluents ranging from 30 to 2,000 ng/mL^17^. Furthermore, it is difficult to sterilize the entire drinking water distribution system. Bacteria may enter the pipe network, attach to the pipe wall and become part of a biofilm. Such bacteria can pose a potential threat to public health if endotoxins are produced.

Although the complex amphiphilic molecular structures of endotoxins vary among different bacterial strains, endotoxins share a common architecture typically consisting of three components, lipid A, core oligosaccharide and O-antigen polysaccharide. The polysaccharide chain is covalently bound to a lipid region, termed “lipid A” by Westphal and his associates^18^. Generally, the structure of the hydrophobic glucosamine-based lipid A is conserved during the biochemical synthesis^19, 20^ and the lipid A part is responsible for the biological activity and toxicity of the LPS. The core polysaccharide region, which is usually identical for large groups of bacteria, consist of two or three 2-keto-3-deoxyoctulosonic acids (KDOs) and 8–15 monosaccharides^21^. The O-antigens, which is responsible for its antigenicity and serotype-specific immunogenicity, are composed of various numbers of repeating oligosaccharide subunits, due to different biosynthesis^19^. Endotoxin monomers have a molecular weight of approximately 10 to 20 kDa. However, due to its amphipathic structures, LPS molecules have strong tendencies to form aggregates into vesicles in aqueous solution with a micellar weight around one million daltons^22^. The smooth-type LPSs, containing all three components in the structure, usually can be isolated from the wild-type bacterial strains. The absence of the O-specific region (due to mutation) results in a shortened and more hydrophobic bacterial strain, with a “rough” colony morphology, are normally called rough-type LPSs^21^.

There are two methods recognized by the Food and Drug Administration (FDA) for determining intact LPSs. They are rabbit pyrogen test (US Pharmacopoeia rabbit test) and Limulus amoebocyte lysate test (LAL-test)^3, 22^. The mechanisms for both rabbit pyrogen test and LAL test are attributed to the biologically reactive lipid A region, which is regarded as the “endotoxic” component of LPS and is responsible for the biological effects^9^. The *in vivo* rabbit pyrogen test suffers from ethical and economic problems^22^ and has gradually been replaced by the *in vitro* LAL test^22^, which is now the most popular method for the analysis of endotoxins. Limulus amoebocytes (cells found in the hemolymph of the horseshoe crab) can form an intracellular gel in the presence of endotoxins and turn the reaction medium yellow in the kinetic chromogenic LAL assay. Although LAL assay is extremely sensitive with detection limit as low as 100 picograms per milliliter^23^, it shows many shortcomings as well^3^. For example, temperature, pH, ions, and even reagent chemicals can influence the LAL results^3^. Besides, the non-specific methods would also give false positive results due to reactions with other microbial products that trigger the LAL reaction, such as peptidoglycan from gram-positive organisms, release of (1-3)-β-D-glucans^9^, and D-glucose polymers from cell walls^24^. Furthermore, the LAL method only provides the general activity of endotoxins in the sample, instead of the detailed structure or distribution profile^25^. LAL method measures endotoxin concentration indirectly, but endotoxins that possess diverse structures and originate from different bacterial serotypes and their mutants may introduce variations. These structural differences will directly affect the endotoxin reactivity and toxicity analyses in the LAL test^26^. Moreover, endotoxins have the ability to form agglutinate or micelles^27^. These micellar structures reduce their reactivity relative to LAL, rendering inaccurate LAL results in reflecting the true concentrations of endotoxins. A reliable analytical method for endotoxin analysis will serve as an useful tool in the monitoring of drinking water purification processes and water reclamation plants.

Other methods have been explored for quantifying endotoxins, such as enzyme-linked immune sorbent assay^28^, gas chromatography coupled with mass spectrometry (GC/MS)^29^ and capillary electrophoresis (CE)^30, 31^. Among these techniques, CE provides rapid and highly efficient separation, and hence is especially suitable for the analysis of large molecules^32^. The analysis of LPS, either after O-deacylation or mild acid hydrolysis, has received considerable attention. However, to our knowledge, the application of CE with LIF detection for the analysis of intact LPS molecules has not previously been extensively studied. Given that LPS lack natural optically active groups in the chemical structure, detection by the commonly used UV absorbance detector proves challenging. Freitag and coworkers^33^ reported a CE with indirect UV-detection method, using a strongly UV-active buffer to obtain “negative” LPS peaks. However, the sensitivity of the UV absorbance detector is limited. Therefore, LIF detectors, being more sensitive by at least three orders of magnitude compared to the UV absorbance detection, are preferable for detecting trace amount of LPS^34^. Since LPS does not possess native fluorescence properties, chemical derivatization with a fluorescent dye is required. Fluorescein isothiocyanate Isomer 1 (FITC) is one of the most widely used fluorescent derivatizing agent for the analysis of amino-group containing analytes, primarily due to its high quantum yield (0.92).

For accurate, reliable and robust analysis of the endotoxins levels in water samples in complicated matrices, a sample preparation method is necessary. Few reports and commercial products were available so far. The sample preparation method serves mainly two purposes. Firstly, the sample pretreatment should be suitable for many different sample matrices. As a result, the pretreatment method in this study can be used to extract the targeted compounds, i.e. endotoxins, from other interference materials. Secondly, it will be helpful to enrich endotoxins during the preparation of the final injection solutions to increase the sensitivity of the analytical method. Solid phase extraction (SPE) can be employed to eliminate the influence of matrix components in real samples. So far, SPE study for endotoxins has not been extensively investigated. This work aimed at developing analytical methods for separating and determining endotoxins in water samples by CE coupled to laser induced fluorescent detection (LIF). In combination with the solid phase extraction method, the CE separation method could be used to determine endotoxin concentrations in both simple water matrices and complex water matrices.

## Results and Discussion

### The labeling of FITC and LPS

FITC is an amine-reactive fluorophore, which reacts with primary and secondary amino groups to form thiourea. This reaction is sensitive to pH and temperature and the optimum reaction condition is reported to be at pH 9.0, and 20–25 °C^35^. The derivatization reaction normally takes place for more than 12 hours due to the extremely slow kinetics.

Earlier studies had confirmed the presence of free amino groups in the form of ethanolamine and 4-amino-4-deoxy-L-arabinose residues (3 deoxy-α-D-mannooctulosonic acid, i.e. KDO) in both the core region and lipid A portion of the LPS molecule^36–38^. Although these amino groups appear to be present in sub-stoichiometric amounts, they make covalent bonding with FITC possible. Furthermore, since the lipid A and the core domain show the greatest similarity among various bacterial strains, labeling this part of the LPS molecule would constitute the most general strategy for LPS detection.

### Optimization of electrophoretic conditions

Influence of several parameters, including the composition, concentration and pH value of the running buffer, the length of the capillary were investigated in order to find the optimal separation conditions.

#### Choice of separation buffer systems

The choice of the separation buffer plays an important role in the electrophoretic patterns of the analytes. Since the most commonly used buffers for the electrophoretic separation of carbohydrates are borate, this could also be applied for the separation of the LPS. Despite the fact that LPSs are neutral compounds, which makes them unsuitable for electrophoretic analysis, complexation of borate ions (B(OH)_4_^−^) with cis-diol groups in the carbohydrate moiety within the LPS structure would enhance the electrophoretic mobilities of LPS^39^. Borate complexation provides the uncharged glycolipid with negative charges thus making them mobile in CE analysis^40^.

Since labeling reagent (i.e. FITC) is an amine reactive fluorophore, any buffer or buffer additives that contain amino groups such as Tris(hydroxymethyl)aminomethane (tris), Tricine and 1,4-diaminobutane (DAB), were tested and ruled out due to sensitivity loss and broad peak. Using borate buffer, separation performance of micellar electrokinetic chromatography (MEKC) with SDS (ranging from 10–40 mM) as surfactant was investigated. However, tailing peaks were observed and worse resolution of background FITC peaks with endotoxins was observed (Figure S1). There might have affinity effects between endotoxins and the micelle cavity or carbohydrate moiety of SDS, but the negative charge brought by SDS made the separation more difficult. Therefore, separation buffer without SDS additive was selected.

#### Effect of buffer concentration

**Figure 1:**
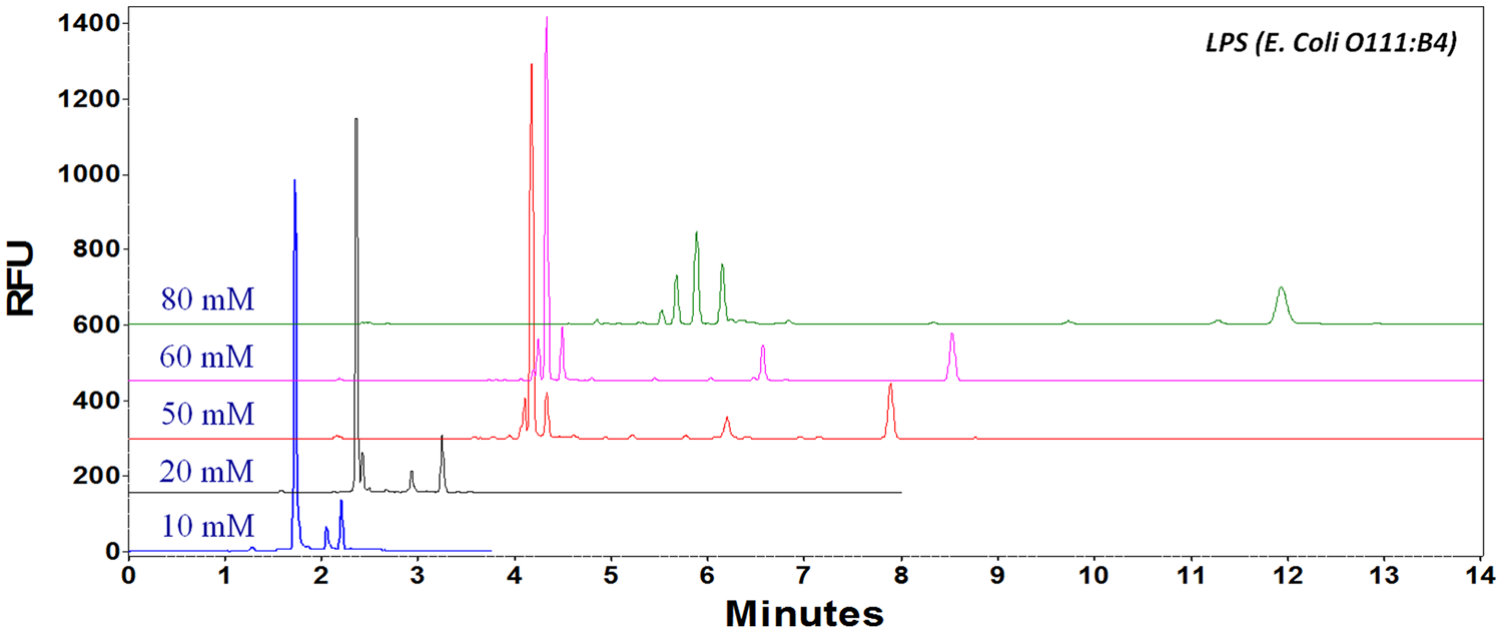
FITC-LPS (from *E. Coli* O111:B4) separated in Na_2_B_4_O_7_ buffer of different concentrations (**1**) 10 mM; (**2**) 20 mM; (**3**) 50 mM; (**4**) 60 mM; and (**5**) 80 mM. Other conditions: Bare fused silica capillary, (I.D. 50 µm). Total length: 39 cm (effective length: 29.0 cm); Voltage: 30 kV; Sample storage: 25 °C; Sample injection time: 5 s.

The effect of sodium tetraborate concentration on the separation efficiency was evaluated in the range of 10–80 mM. In Fig. 1, small FITC-labeled LPSs migrated out first, followed by large background peaks from more negatively charged free FITC, which was in excess amount. By increasing the buffer concentration, the ionic strength increased, and thus migration time increased due to the decreased EOF. Although lower buffer concentrations (i.e. 10 or 20 mM) gave shorter separation times, peaks were not well separated. With high buffer concentration (80 mM), fluorescence intensities of FITC labeled LPS dramatically decreased due to Joule heating effect caused by higher current and peak diffusion caused by longer separation time. Consequently, 50 mM sodium tetraborate was chosen as the optimum buffer concentration, which gave the highest fluorescence intensity and satisfactory resolution.

#### Effect of buffer pH and capillary length

**Figure 2:**
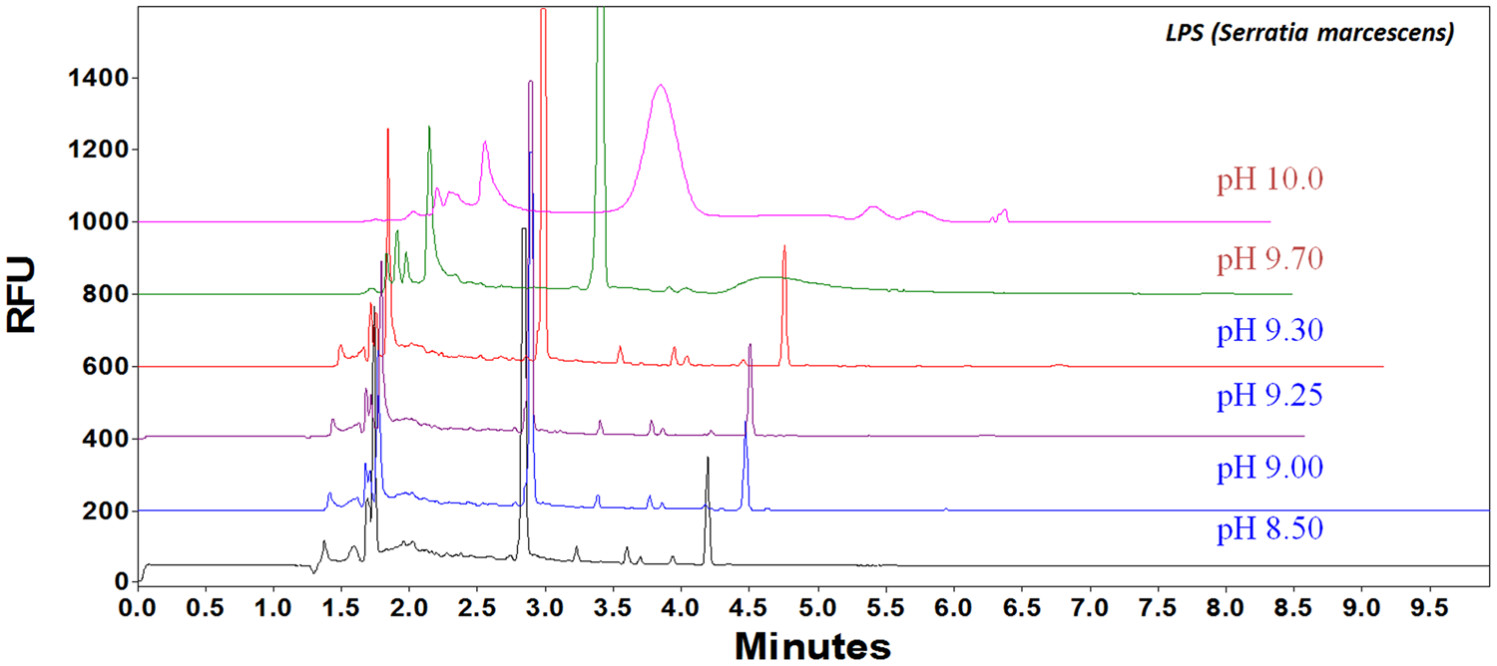
FITC-LPS (from *Serratia marcescens)* separated in sodium tetraborate buffer (50 mM) with different pH values: (**1**) 8.50; (**2**) 9.00; (**3**) 9.25; (**4**) 9.30; (**5**) 9.70; and (**6**) 10.0. Other conditions: Bare fused silica capillary (I.D. 50 µm); Total length: 39 cm (effective length: 29.0 cm); Voltage: 30 kV; Sample storage: 25 ^o^C; Sample injection time: 5 s.

It has been reported that in a pH range from 8 to 12, carbohydrates could be associated with a negative charge, and thus borate complexation would take place with negatively charged LPS^40^. Consequently, the effect of pH value of the separation buffer was investigated by varying the pH of the separation buffer from 8.50 to 10.0. Better resolution and longer migration times of analytes were observed (Fig. 2) by increasing the pH of the running buffer. However, broadened peaks appeared at pH values of 9.70 and 10.0. The reasons might be due to FITC degradation or higher ion strength introduced during pH adjustment. Thus, pH 9.30 was selected to achieve a compromise between the resolution of the analytes and the separation time.

Another parameter studied was the length of the capillary. As showed in Figure S2, the shortest capillary of 38.8 cm (effective length of 29.0 cm) allowed a reduction in analysis time (within 10 min) while still offered a clear separation of LPS peaks from FITC background, and thus was selected to be the optimal capillary length. Using the optimized conditions, FITC labeled LPS peaks could be separated, and detected at around two minutes.

### Detection of different LPSs by CZE

**Figure 3:**
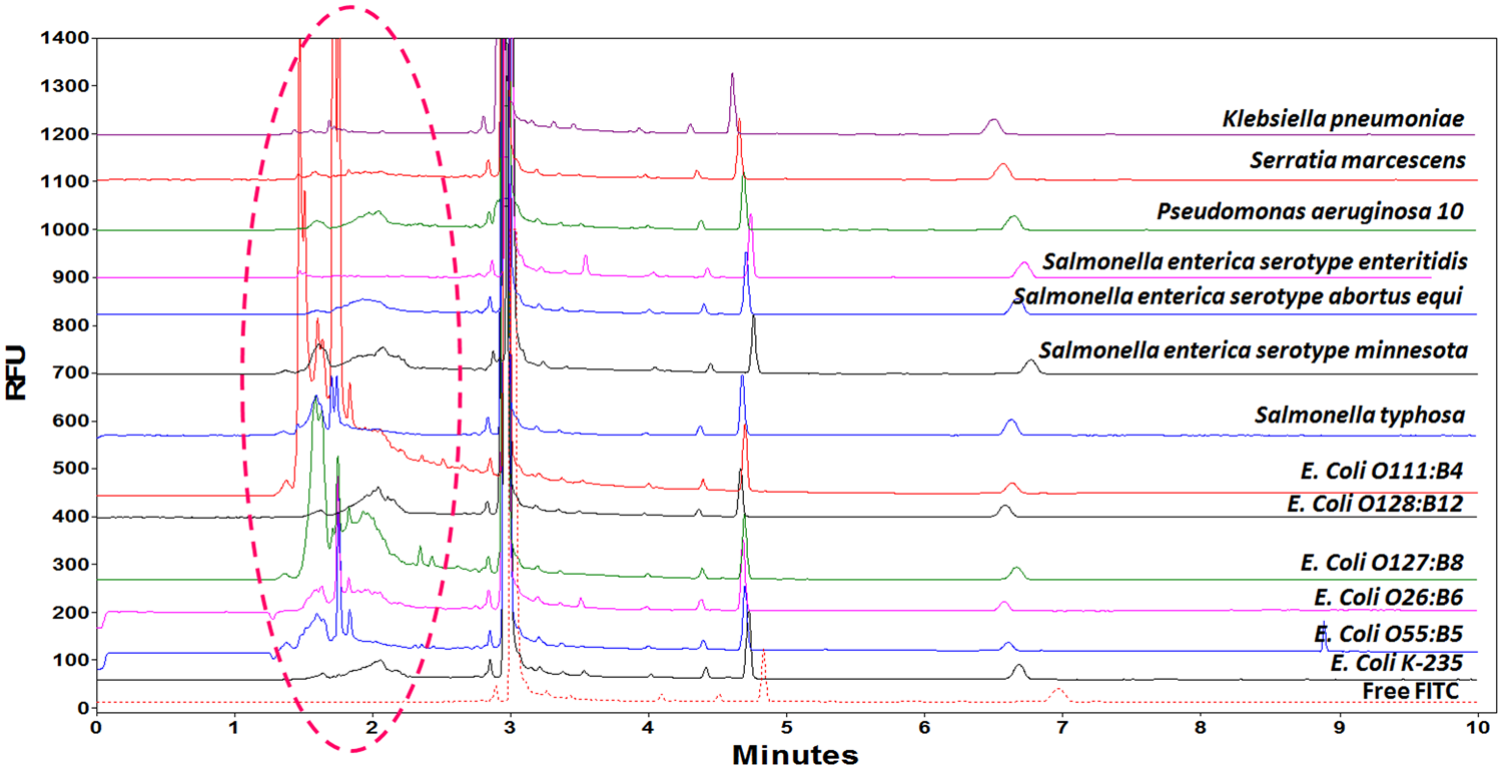
CE-LIF separation of 13 endotoxins. LPS concentration: 500 µg/mL. Separation in buffer: Na_2_B_4_O_7_ (50 mM), pH 9.30; Bare fused silica capillary, (I.D. 50 µm); Total length: 39 cm (effective length: 29.0 cm); Voltage: 30 kV; Sample storage: 25 °C; Sample injection time: 5 s.

The movement of molecules in capillary electrophoresis is based on their mass charge ratio. Figure 3 shows the CE separation of 13 LPS standards under the optimized conditions. The electropherograms of the LPS standards exhibit distinctive individual patterns. These observations are due to the LPS standards’ mass charge ratios and the different structural properties in the respective components of these standards. However, there are no remarkable differences in the migration times. All the FITC-LPS peaks were detected at around 2 min.

As there are many different bacteria strains in the environment and therefore numerous possibilities of LPS sources, it is impossible to confirm which bacteria generates certain particular LPS. Instead, it is more realistic to measure all LPSs and provide an estimated total LPS level, in a similar way as previous analytical methods of LPS, such as LAL. Since LPS from *Escherichia Coli O55:B5* (a standard strain - ATCC 12014) is commonly used as the standard endotoxins in LAL assay, LPS from *Escherichia coli O55:B5* was also used for quantification by the CE-LIF method as reference value. Endotoxin levels were calculated relative to the level of the reference endotoxin.

To study the accuracy of quantification of LPS, a calibration curve was established using LPS from *E. Coli O55:B5* with 12 different LPS concentrations in the range of 0.05 µg/mL to 100 µg/mL. For each concentration seven measurements were carried out. Good linear correlation was obtained with R^2^ = 0.9991. The LOD is about 50 ng/mL at a signal to noise ratio (S/N) of 3.

The stability of the derivatized LPS molecules in aqueous phase was also investigated. The stability test was carried out during a period of 20 days using LPS from *E. Coli O55:B5*, with the samples stored in dark at room temperature for 20 days. The samples were analysed each day to obtain consecutive electropherograms. The results showed that FITC itself was not very stable in aqueous solution at room temperature as a significant decrease in fluorescence signal was observed with longer storage times. In contrast, after labelling with LPS, the product exhibited high stability in aqueous solution at room temperature. No obvious degradation was found after 20 days (Figure S3) of storage at room temperature. So the FITC labelling method should have great robustness in real analytical work.

### Solid phase extraction method to purify and enrich endotoxin

The selectivity of the stationary phase is an important parameter to be considered when the analytes are to be extracted from water. There are a large number of solid phase extraction materials available commercially, covering a wide range of selectivity and applications. The primary decision for trace analysis of endotoxin is the selection of the proper type of sorbent. Silica-based aminopropyl sorbent has the capacity to strongly and selectively retain analytes that are hydrophilic in nature. Specifically, the retention of carbohydrates onto a HILIC stationary phase can be explained in terms of the hydrogen bonding as well as ionic and dipole-dipole interactions that occur while the carbohydrates partition into an immobilized water layer. As carbohydrate is one of the main components in the LPS structure, which is hydrophilic, the aminopropyl sorbent should be applicable to retain LPS.

#### Smooth type LPS extraction

**Figure 4:**
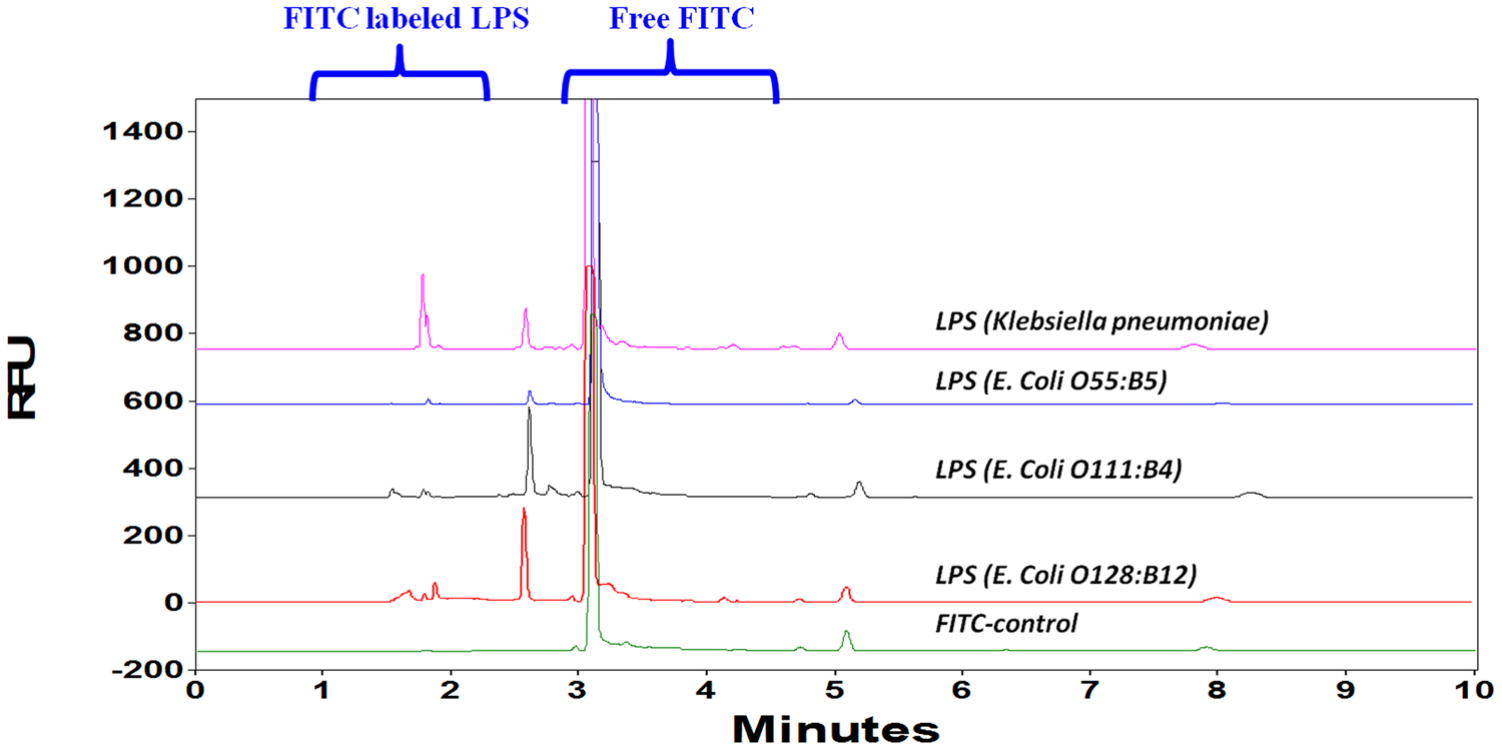
Analysis of FITC labelled LPS extracted by Waters HILIC µElution plate. Separating in buffer: Na_2_B_4_O_7_ (50 mM), pH 9.30; Bare fused silica capillary, (I.D. 50 µm). Total length: 39 cm (effective length: 29.0 cm); Voltage: 30 kV; Sample amount: 100 µL; Sample storage: 25 °C; Sample injection: 5 s.

**Figure 5:**
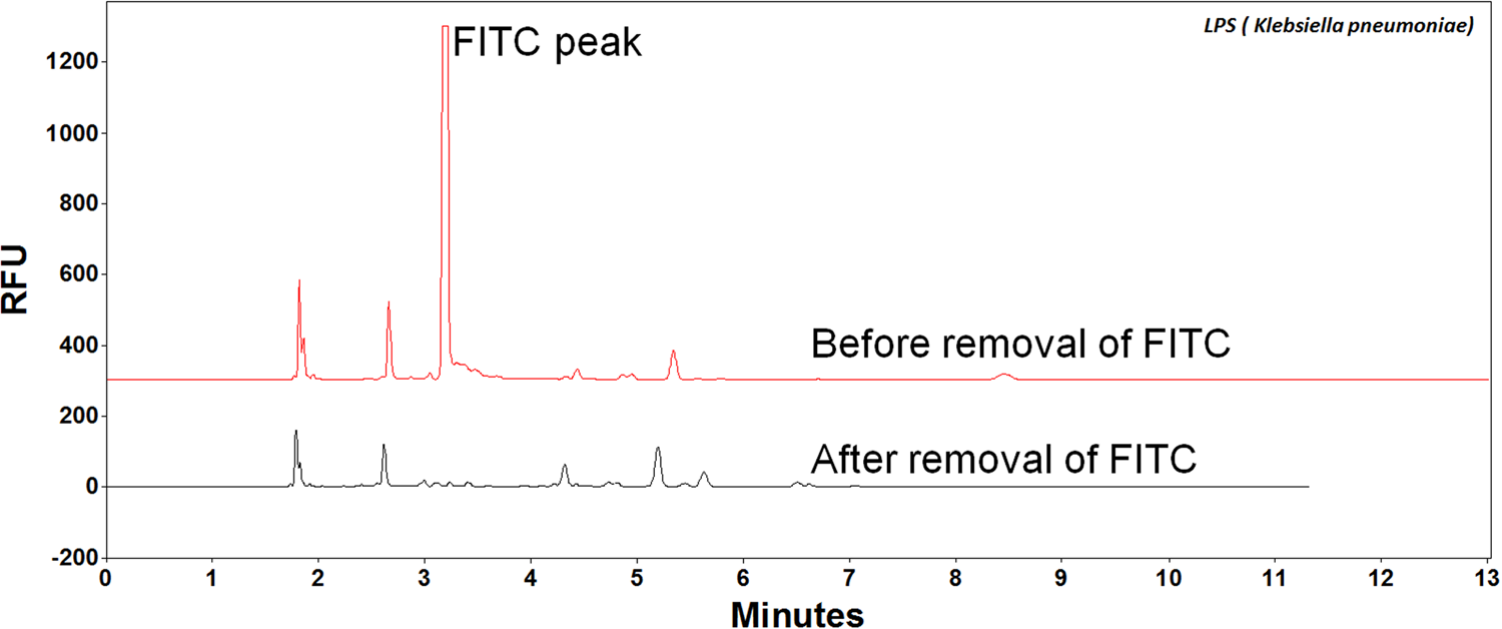
HILIC µElution plate to remove the excess FITC dye from labeled LPS from *Klebsiella pneumoniae*. Separating in buffer: Na_2_B_4_O_7_ (50 mM), pH 9.30; Bare fused silica capillary, (I.D. 50 µm). Total length: 39 cm (effective length: 29.0 cm); Voltage: 30 kV; Sample storage: 25 °C; Sample injection: 5 s.

Since the fluorescent dye FITC targets the amino groups in lipid A and the core region, solid-phase extraction (SPE) method specifically designed for capturing polysaccharides can remove most interference substances and cross-validate the analytical method. For example, peptides or proteins can be labeled by FITC, but they will be removed during the SPE step; whereas polysaccharides can be captured by SPE, but they will not be labeled by FITC and therefore will not be detected by the CE-LIF method. Figure 4 indicated that LPS peaks were preserved after the SPE procedure. It could be confirmed further by comparing the LPS concentrations in different step eluents. Only the final elute showed significant endotoxin peak. Moreover, the SPE procedure was able to not only purify and enrich endotoxins, but also to remove excess fluorescent dyes after the labeling process (Fig. 5). The large FITC background was removed after SPE.

It is worth noting that the SPE pretreatment and CE-LIF method could prevent possible false positive results commonly associated with LAL assays that are due to the reaction with (1-3)-β-D-glucans as these interference substances will trigger the LAL reaction. In contrast, the CE-LIF method provides inherent discrimination of interfering substances including (1-3)-β-D-glucans (Figure S4), due to the specificity of the derivatizing agent.

#### Rough type LPS extraction

**Figure 6:**
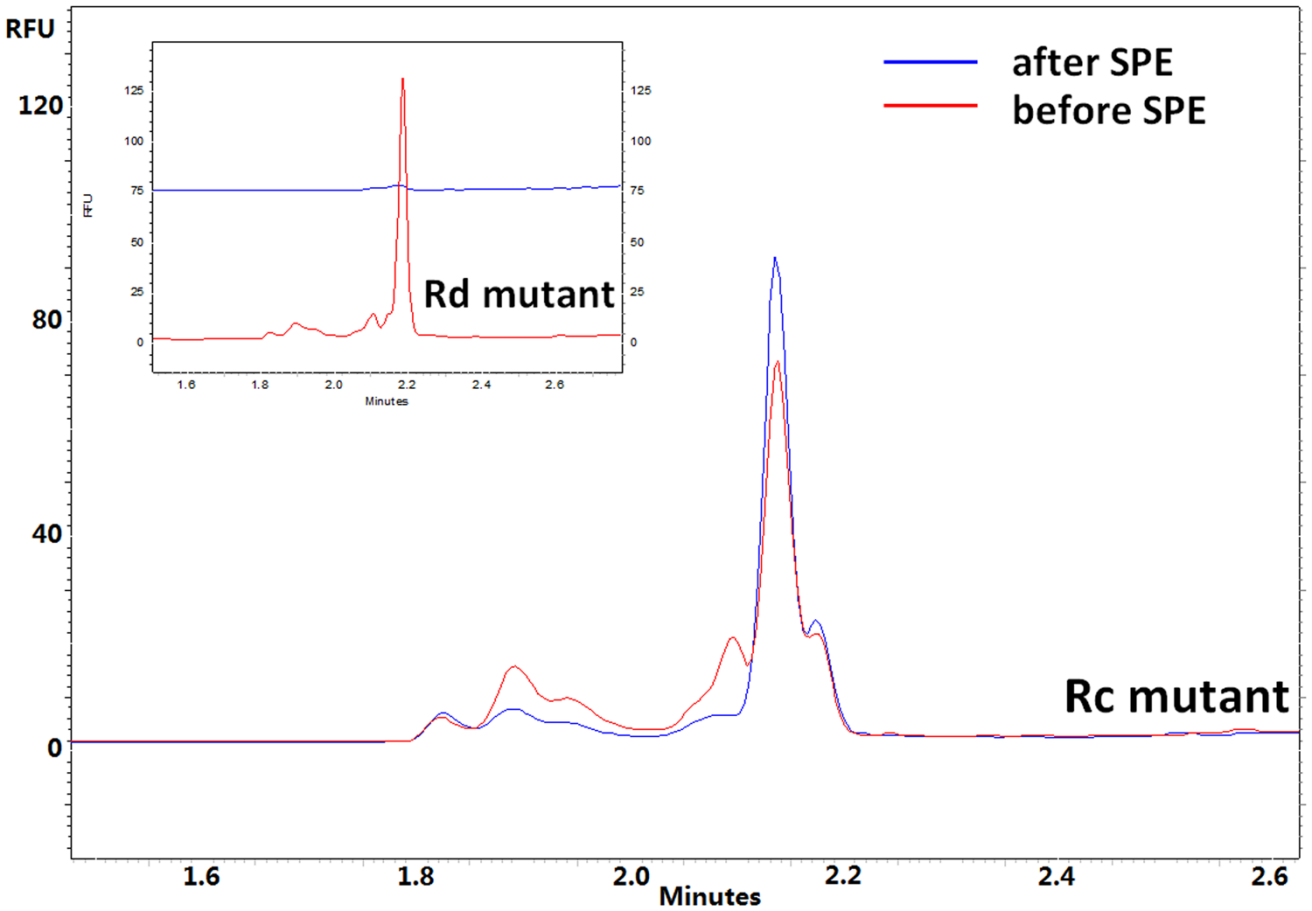
Loss of short R-type endotoxin with SPE extraction procedures (If oligosaccharide was shorter than Rc mutant). CE conditions: Separation buffer: Na_2_B_4_O_7_ (50 mM), pH 9.30; Bare fused silica capillary, (I.D. 50 µm); Total length: 39 cm (effective length: 29.0 cm); Voltage: 30 kV; Sample storage: 25 °C; Sample injection time: 5 s.

As SPE targeted at the carbohydrate part of LPS, it is reasonable to note the limitation of this method, because LPSs with very short oligosaccharide chains may be lost during the SPE steps. Most wild strains of gram-negative bacteria can generate smooth type LPS. However, mutations of the wild strain bacterium can be induced by UV light or exposure to other mutagenic-causing compounds, resulting in R-type LPS with shorter polysaccharide chains. Ra, Rb, Rc, Rd and Re denote the different rough chemotypes of LPS, while each alphabetic increase means loss of several monosaccharides on the carbohydrate chain. Re-type LPS actually would have lost all O-antigen moiety and only core-oligosaccharide remains. The extreme case of Re-type LPS consists of lipid A carrying only two KDO residues. To investigate the applicability of the SPE method to LPSs with different lengths of carbohydrate chains, several R-type LPSs with shorter carbon chains were tested. The results indicated that the SPE method was suitable for extracting LPS with Rc or longer carbohydrate chains. In contrast, shorter carbohydrate chain LPSs may be lost during SPE as the Rd mutant LPS peak became very small after SPE (Fig. 6). Nevertheless, it can be concluded that the SPE protocol is suitable for the analysis of the vast majority of endotoxins in nature.

It is also interesting to observe the long migration times for R-type LPSs in Fig. 6. In contrast, all LPS peaks in Fig. 4 were observed before 2 min. As R-type structures have shorter carbohydrate chains and therefore larger charge-to-size ratios, it is reasonable to infer that migration times of LPSs can be used to distinguish the S and R types of LPS.

#### Quantitative data of SPE and CE-LIF combination

Calibration was performed for LPS standard (from *E. Coli* O55:B5) in the range of 0.05–100 µg/mL. Combining SPE extraction and CE separation, the whole protocol showed good linearity (R^2^ = 0.9984). Analysis of 50 ng/mL LPSs from *E. Coli O55:B5* and *E. Coli O26:B6* by CE-LIF demonstrated good repeatabilities, with RSD of peak area at 2.82% and 2.94%, RSD of migration time at 0.33% and 0.40%, respectively (n = 6). Six LPS standards were subjected to six different SPE columns, and then the eluents were labelled with FITC and introduced into the CE system for analysis. The peak area RSD values acquired with the SPE procedure were 8.44% and 11.0% for LPSs from *E. Coli O55:B5* and *E. Coli O26:B6*, respectively.

As the SPE method possessed sample enrichment capability, larger sample volumes were tested to confirm this effect. LPS from *E. Coli O26:B6* showed 83.4% recovery when sample volume was increased to 1 mL, while the recovery using 100 µL sample volume was 89.9%, indicating that an enrichment factor of 10 was achievable with the SPE pre-treatment and CE-LIF protocol. However, the extraction device might not suitable for larger volume sample extraction, as the recovery dropped to 58.6% and 54.8% for volumes of 3 ml and 10 ml respectively. Meanwhile, the present results showed accurate resolution of LPS peaks from other interferences, so it is possible to increase sensitivity by increasing sample injection time and using 100 µm ID capillary, which may theoretically provide 4-times sensitivity increase. After dilution by ultrapure water, the LOD of endotoxin using 100 µm ID capillary could reach around 5 ng/mL as shown in Figure S5. The noisy baseline could be attributed to ubiquitous bacterial endotoxins in the environment. Therefore, if all reagents and apparatus are strictly pyrogen-free, the detection limit of the whole method would be able to reach around 1 ng/mL, which is comparable with typical LAL assay.

### Determination of LPS concentration in water samples

**Figure 7:**
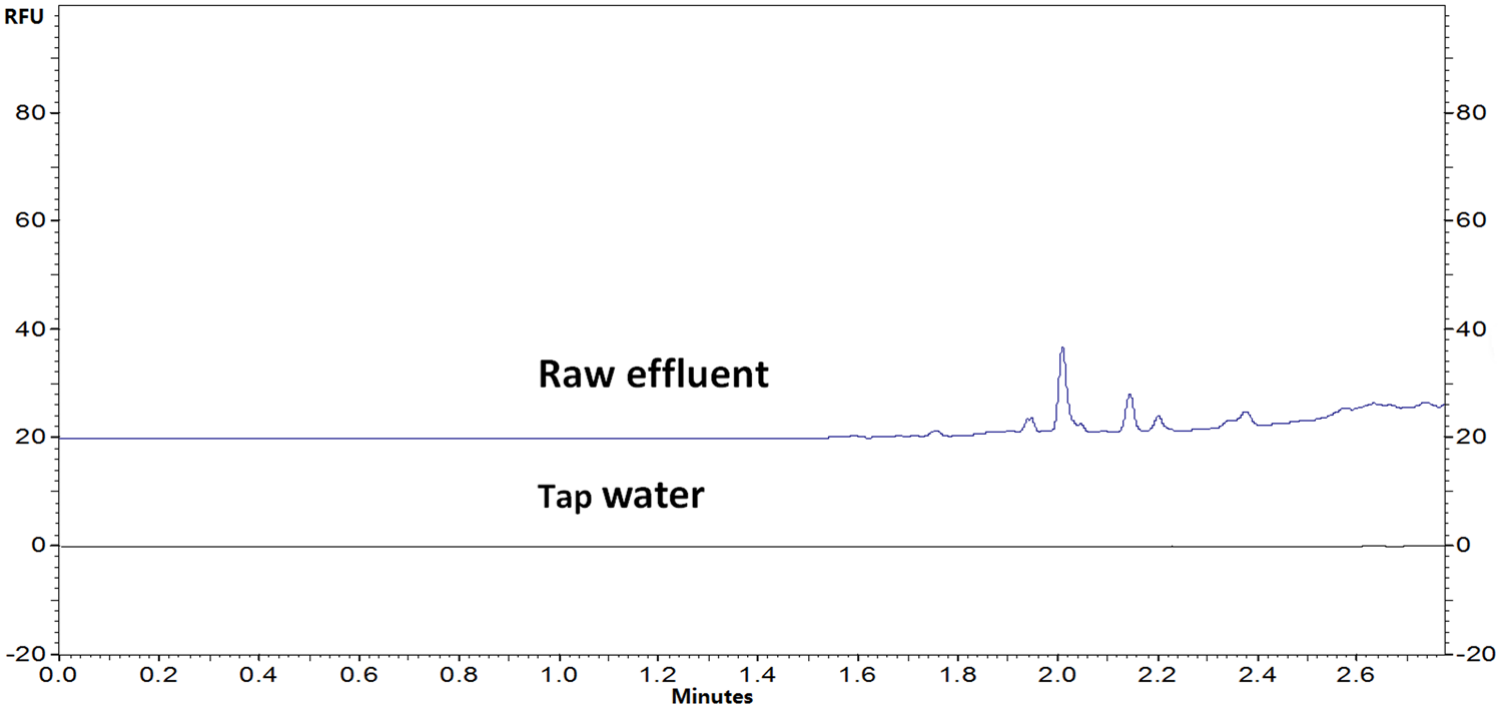
Analysis of LPS level in raw effluent and tap water. CE conditions: Separating in buffer: Na_2_B_4_O_7_ (50 mM), pH 9.30; Bare fused silica capillary, (I.D. 50 µm); Total length: 39 cm (effective length: 29.0 cm); Voltage: 30 kV; Sample storage: 25 °C; Sample injection time: 5 s.

To demonstrate the applicability of the method developed in real sample analysis, determination of LPS in water samples were performed using the optimum conditions. The water samples include tap water and raw effluent before wastewater treatment. All the samples were stored at 4 °C before FITC derivatization and CE-LIF analysis. Several LPS peaks were clearly observed for the raw effluent sample in Fig. 7, which corresponded to LPS level of 3.79 mg/mL. In contrast, no clear LPS peak was observed for tap water sample.

Furthermore, spike-and-recovery experiments were then carried out in order to investigate the accuracy of the SPE pre-treatment and CE-LIF method. Known amounts of LPS standard (from *E. Coli* O26:B6) were added to the raw effluent water sample, and the resulting spiked samples were subjected to SPE and derivatization procedures, and eventually subjected to CE-LIF analysis. Recoveries were calculated based on the calibration equation and the differences between the calculated concentration in the spiked samples and the known original concentrations. The recoveries determined for spiking 20 ppm and 60 ppm LPS standards were 105.0% and 83.4%, respectively. These results showed that the proposed SPE pretreatment and CE-LIF method could be used for identification and quantification of LPSs in water samples. To validate the repeatability of our testing methods, both inter- and intra-day analyses were conducted to ensure robustness of our method (Table 1). At LPS concentration of 13.02 mg/L, our intra-day tests reported low variance in migration time (RSD = 0.56%; *n* = *6*) and peak area (RSD = 4.24%; *n* = *6*). For the inter-day tests (3 days, *n* = *6*), we report low standard deviations in both the migration time (average RSD = 0.44%) and peak area (RSD = 3.6%). Moreover, accuracy tests were executed to ascertain the calibration variance of our protocol. For the LPS standard concentration of 13.02 ppm, we obtained 6 readings (intra-day, *n* = *6*, RSD = 4%) that gave us the desirable accuracy range of up to 91.3%. For the inter-day tests, our three-day tests yielded concentrations of standards with average RSD of 2.28%.

**Table 1:** Reproducibility of LPS Concentration Measurements.

**Parameter**	**Average RSD (%)**
Intra-day repeatability (n = 6)	
Migration time	0.56
Peak area	4.24
Measured concentration^^^	4.00
Inter-day repeatability (n = 3 days)*	
Migration time	0.44
Peak area	3.6
Measured concentration^#^	2.28

*Based on 6 replicates on each day.

^^^Based on known concentration of 13.02 ppm, measured mean = 11.40 ppm.

^#^Based on known concentration of 13.02 ppm, measured mean = 11.23 ppm.

## Conclusion

In this paper we demonstrated a novel protocol for endotoxin analysis, by using HILIC µElution SPE plate to extract endotoxins from water samples, followed by pre-column FITC derivatization and a CZE separation coupled with LIF detector. It is well known that the identification and characterization of intact LPS, as amphiphilic macromolecular lipoglycan, is a complex and challenging area of research. One of the major problems is due to the heterogeneity of the LPS structure among various bacterial species and strains, as well as the inert properties of the structure. Our method is focused on the common structure that almost every LPS molecule share, so that it has the versatility to detect LPS from almost all bacterial species and strains. Besides, compared with the widely used LAL test, this method provides inherent discrimination of interfering substances including (1–3)-β-D-glucans, due to the specificity of the derivatizing agent. Furthermore, in this study, the combination of SPE extraction methods and the FITC labeling strategy furnishes significant advantages in terms of selectivity and sensitivity of the analysis method. The developed CE-LIF method has been tested with environmental water samples, and demonstrated sensitive, reproducible and reliable results.

## Methods

### Chemicals

Sodium dodecyl sulfate (SDS), sodium tetraborate, and sodium hydroxide were obtained from Merck (Darmstadt, Germany). Fluorescein isothiocyanate (FITC), (1-3)-β-D-glucans were purchased from Sigma - Aldrich (St. Louis, MO, USA). Lipopolysaccharides from *Escherichia coli* 0111:B4 (Lot # 024M4019V), *Escherichia coli* 055:B5(Lot #025M4040V), *Escherichia coli* 026:B6 (Lot # 053M4060V), *Escherichia coli* 0127:B8 (Lot # 103M4051V), *Salmonella enterica* serotype enteritidis (Lot # 064M4035V), *Pseudomonas aeruginosa* 10 (Lot # 100M4101V), *Salmonella enterica* serotype typhimurium (Lot # 093M4088V), *Escherichia coli* J5 (Rc mutant, rough strains) (Lot # 053M4112V), *Salmonella typhosa* (Lot # 063M4017V)*, Escherichia coli* F583 (Rd mutant, rough strains) (Lot # 055M4005V), *Salmonella enterica* serotype minnesota (Lot # 064M4015V), *Escherichia coli* 0128:B12 (Lot # 063M4014V), *Klebsiella pneumoniae* (Lot # 111M4038V), *Escherichia coli* K-235 (Lot # 031M4076V), *Salmonella enterica* serotype abortusequi (Lot # 104M4064V), and *Serratia marcescens* (Lot # 013M4078V) were purchased from Sigma Aldrich (St. Louis, MO, USA). Buffers were prepared with ultrapure water obtained from Merck Millipore (Millipore, Bedford, MA, USA). All other reagents were of analytical grade. Experiments were carried out using freshly prepared and filtered solutions.

### Instrumentation

All electrophoretic separations were performed on a Beckman Coulter PA 800 plus system (AB Sciex, Concord, Ontario, CA), equipped with a solid-state laser induced fluorescence detector with excitation wavelength of 488 nm and emission wavelength of 520 nm. The 32 Karat version 9.1 software was used for data analysis, data acquisition and peak integration.

### Labeling LPS with FITC

LPS was labeled with FITC by a protocol similar to that of Skelly *et al*.^41^ with slight modifications. Briefly, FITC was dissolved in 50 mM sodium tetraborate buffer (pH 9.30). LPS solution was prepared by dissolving LPS powder in ultra-pure water. Then, FITC was added to LPS solution in the mass ratio of 2: 1 and incubated in dark at 37 °C overnight. Normally the fluorescent dye is in excess amount to ensure complete labeling of LPS. FITC-labeled LPS was protected against light exposure throughout.

### Solid phase extraction

Solid phase extraction (SPE) was performed using the 96-well MassPREP™ HILIC (hydrophilic interaction chromatography) µElution plate (Waters, Milford, MA, USA) according to the manufacturer’s protocols. Briefly, 100 µL LPS standards or samples were constituted with 90% (v/v) acetonitrile. Then the sorbent was conditioned with 200 µL of HPLC grade water and equilibrated with 2 × 200 µL of 90% (v/v) acetonitrile. LPS samples were loaded on the sorbent without vacuum, followed by sample washing twice with 200 µL of 90% acetonitrile. Finally analytes were eluted with 100 µL of 1 mM Tris-citrate buffer.

### Capillary Electrophoresis

Fused-silica capillary were purchased from Polymicro Technologies Inc. (Phoenix, AZ, USA). Capillaries with an internal diameter of 50 or 100 µm were used throughout. Prior to use, capillaries were rinsed with 1 M NaOH for 10 min, followed by rinsing with DI water for 10 min and separation buffer for 10 min. The samples were injected into the capillary by pressure injection for 5 s or 10 s under a pressure of 0.5 psi. The system was operated at a constant voltage of 30 kV. The sample storage plate temperature was set to be 16 °C and the separation temperature was set to be 25 °C. Separation buffer was refreshed after every five runs. Between runs, the capillary was conditioned by rinsing with 0.1 M NaOH for 2 min, ultra-pure water for 3.0 min and separation buffer for 5 min.

### Water samples and sample pretreatment

All testing samples were collected in 100 ml glass bottles and were stored at 4 °C until analysis. Before analysis, the water samples were centrifuged at 3000 RPM for 5 min. The solid suspended particles retained at the bottom were discarded and the supernatant was used for analysis. Then SPE was performed using µElution plate. The eluents were labeled with FITC before CE-LIF analysis.

## Electronic supplementary material

Supplementary Information
