# Supplementary material for: Extraction, separation and characterization of endotoxins in water samples using solid phase extraction and capillary electrophoresis-laser induced fluorescence
Source: Sci Rep. 2017 Sep 7;7:10774. doi: 10.1038/s41598-017-11232-x (PMC5589922; doi:10.1038/s41598-017-11232-x)
Supplement: Supplementary file 1 — Supplementary Information [file 41598_2017_11232_MOESM1_ESM.pdf]

Supporting Information

Title: Extraction, separation and characterization of endotoxins in water samples using solid phase extraction and capillary electrophoresis-laser induced fluorescence

Fun Man Fung[1,2], **Min Su**[1], **Hua-tao Feng**[1,3*], **Sam Fong Yau Li**[1,3*]

[1] Department of Chemistry, National University of Singapore, 3 Science Drive 3, Singapore, 117543, Singapore

[2] Institute for Application of Learning Science and Educational Technology (ALSET), University Hall, Lee Kong Chian Wing UHL #05-01D, 21 Lower Kent Ridge Road, Singapore, 119077, Singapore

[3] NUS Environmental Research Institute, National University of Singapore, T-Lab Building, 5A Engineering Drive 1,Singapore, 117411, Singapore

*** Corresponding Author:**

Email: chmlifys@nus.edu.sg. Tel: (65)-6516-2681. Fax: (65)-6779-1691.

Email: chmfeng@nus.edu.sg. Tel: (65)-6516-1953. Fax: (65)-6779-1691.

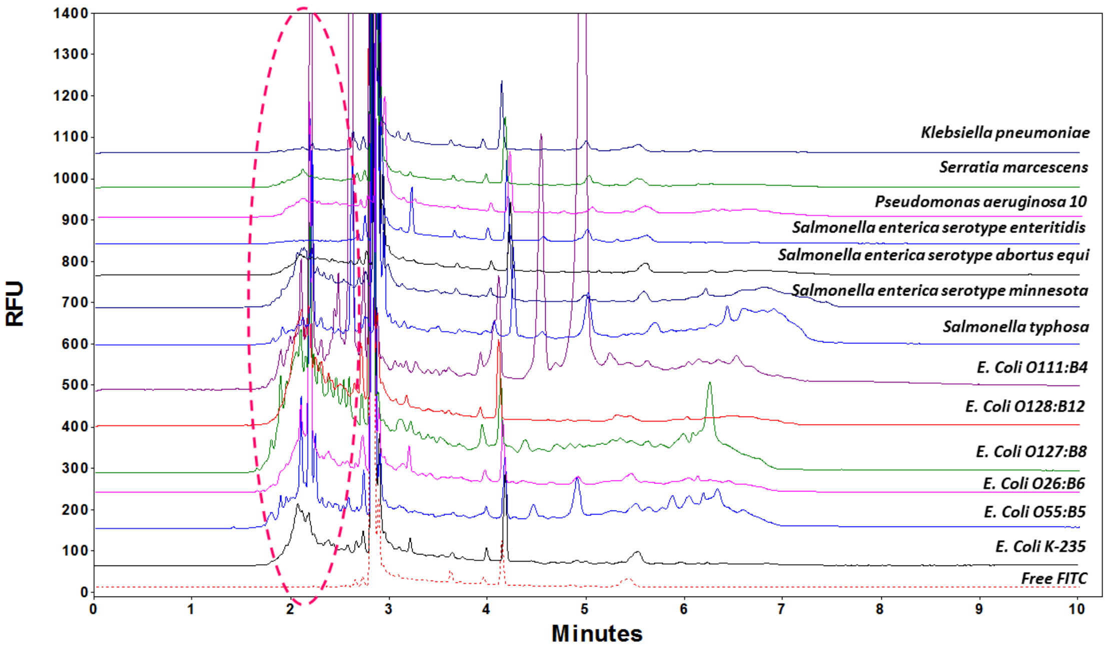

Fig. S1 LPS separation in 40 mM $Na_2B_4O_7$ buffer and 40 mM SDS, pH 9.30; Bare fused silica capillary, (I.D. 50 µm); Total length: 50 cm; Voltage: 30 kV; Sample storage: 25 ^{o}C; Sample injection time: 5 s.

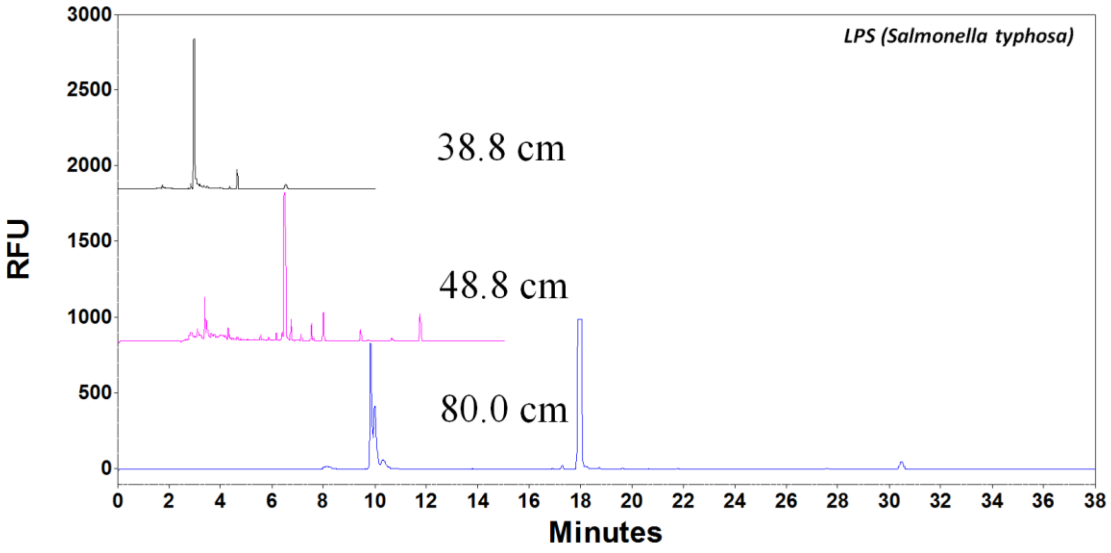

Fig. S2 LPS separation in 50 mM $Na_2B_4O_7$ buffer with different capillary lengths. Other conditions: Separation buffer: $Na_2B_4O_7$ (50 mM), pH 9.30; Bare fused silica capillary, (I.D. 50 µm); Total length: 39 cm; Voltage: 30 kV; Sample storage: 25 °C; Sample injection time: 5 s.

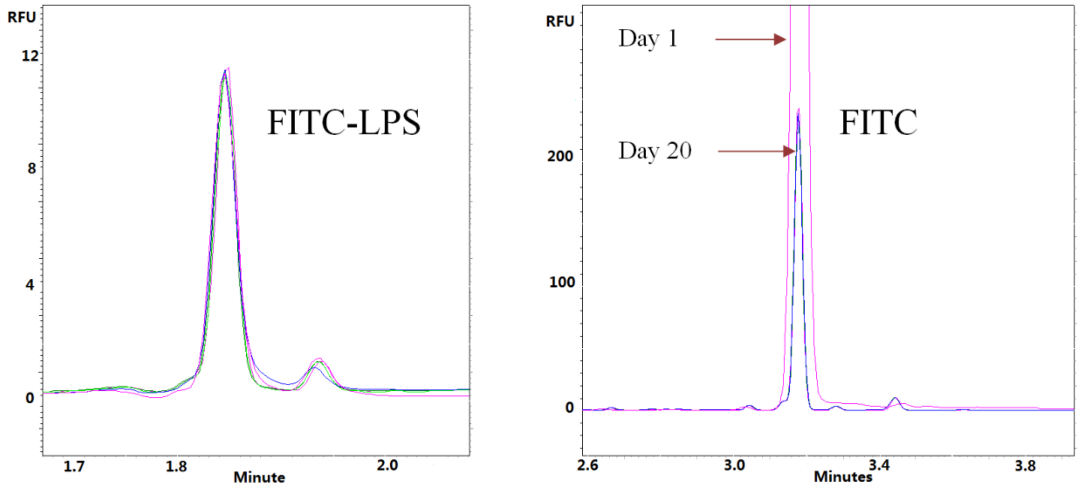

Fig. S3 Stability study of LPS-FITC from Day 1 to Day 20. Other conditions: Separating in buffer: $Na_2B_4O_7$ (50 mM), pH 9.30; Bare fused silica capillary, (I.D. 50 µm); Total length: 39 cm; Voltage: 30 kV; Sample storage: 25 °C; Sample injection time: 5 s.

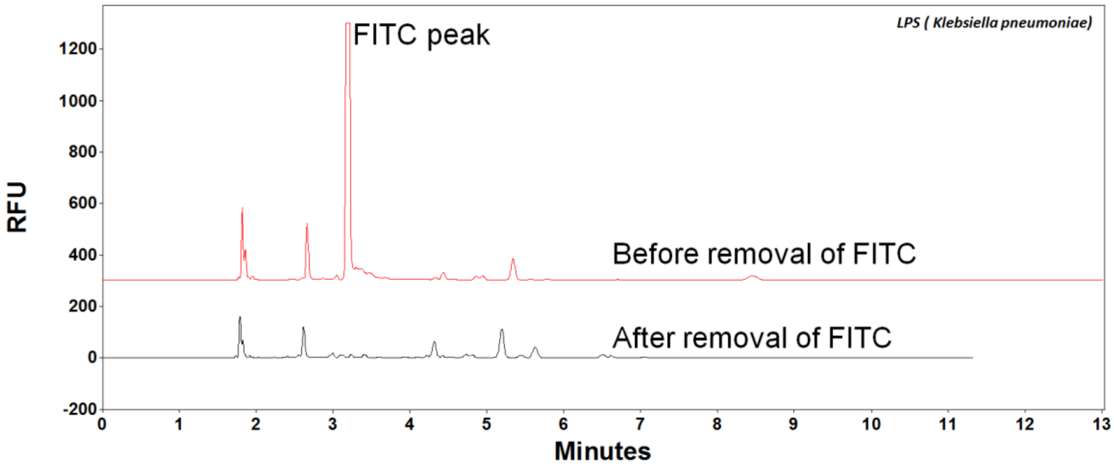

Fig. S4 (1-3)-β-D-glucans interference test. (1) FITC-beta-glucan; (2) FITC control. Other conditions: Separating in buffer: $Na_2B_4O_7$(50 mM), pH 9.30; Bare fused silica capillary, (I.D. 50 µm); Total length: 39 cm; Voltage: 30 kV; Sample storage: 25 °C ; Sample injection time: 5 s.

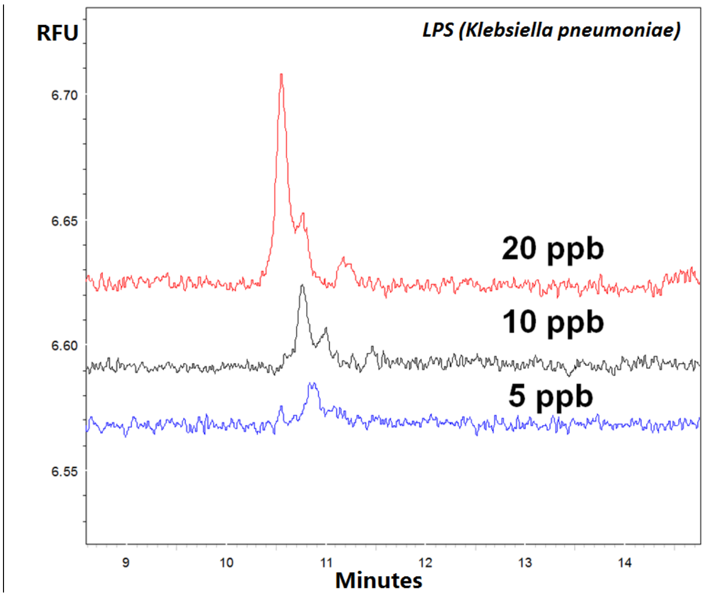

Fig. S5 Investigation of detection limit of endotoxin from *Klebsiella Pneumonia* by ultrapure water dilution. Other conditions: Separating in buffer: $Na_2B_4O_7$ (50 mM), pH 9.30; Bare fused silica capillary, (I.D. 100 µm); Total length: 60 cm; Voltage: 15 kV; Sample injection time: 10 s.
